# Evolutionary rewiring of bacterial regulatory networks

**DOI:** 10.15698/mic2015.07.215

**Published:** 2015-07-06

**Authors:** Tiffany B. Taylor, Geraldine Mulley, Liam J. McGuffin, Louise J. Johnson, Michael A. Brockhurst, Tanya Arseneault, Mark W. Silby, Robert W. Jackson

**Affiliations:** 1 School of Biological Sciences, University of Reading, Whiteknights, Reading RG6 6AJ, UK.; 2 Department of Biology, University of York, Wentworth Way, York YO10 5DD, UK.; 3 Department of Biology, Université de Moncton, Moncton, New Brunswick, Canada.; 4 Department of Biology, University of Massachusetts Dartmouth, 285 Old Westport Road, North Dartmouth, MA 02747, USA.; 5 The University of Akureyri, Borgir vid Nordurslod, IS-600 Akureyri, Iceland.

**Keywords:** bacterial motility, flagella regulation, nitrogen regulation, gene network evolution, enhancing binding proteins

## Abstract

Bacteria have evolved complex regulatory networks that enable integration of multiple intracellular and extracellular signals to coordinate responses to environmental changes. However, our knowledge of how regulatory systems function and evolve is still relatively limited. There is often extensive homology between components of different networks, due to past cycles of gene duplication, divergence, and horizontal gene transfer, raising the possibility of cross-talk or redundancy. Consequently, evolutionary resilience is built into gene networks - homology between regulators can potentially allow rapid rescue of lost regulatory function across distant regions of the genome. In our recent study [Taylor, *et al.* Science (2015), 347(6225)] we find that mutations that facilitate cross-talk between pathways can contribute to gene network evolution, but that such mutations come with severe pleiotropic costs. Arising from this work are a number of questions surrounding how this phenomenon occurs.

Mutational inactivation of two key genes in *Pseudomonas fluorescens*, a peptide synthase (*viscB)*, which is involved in making a surfactant and enabling sliding motility, and the master regulator (*fleQ*) required for flagellum biosynthesis and swarming motility, produced a strain that was immotile on the surface of soft agar despite a perfectly functional flagellum regulon existing in the cell. According to our current knowledge of the flagellum regulatory system, the removal of FleQ from the regulatory system should be a mutation that is catastrophic and irrecoverable in an environment where motility is essential to reach nutrients, like the surface of agar; the mutant should go extinct as it starves to death at the site of inoculation. However, we observed a remarkable outcome: the restoration of motility due to the resurrection of the flagellum. We showed that the evolution of motility followed a rapid and repeatable two-step trajectory in two independent *P. fluorescens* strains by rewiring their nitrogen regulation system to repurpose regulatory activity towards the flagellum regulon (Figure 1). In multiple independent replicates, we found that step 1 mutations could occur in one of three nitrogen genes, *ntrB*, *glnA* or *glnK*. These mutations caused a slow spreading phenotype. A second, faster spreading, mutant then emerged, carrying one of these initial mutations plus an additional mutation in *ntrC*; seven different *ntrC* mutations were found. Our model predicts that each of the step 1 mutations would have increased intracellular levels of phosphorylated NtrC, which then starts activating flagellum genes (seen by microarray analyses); the step 2 mutation then shifted NtrC specificity away from its cognate *ntr* targets and restores *flg* gene expression to wild type levels.

**Figure 1:**
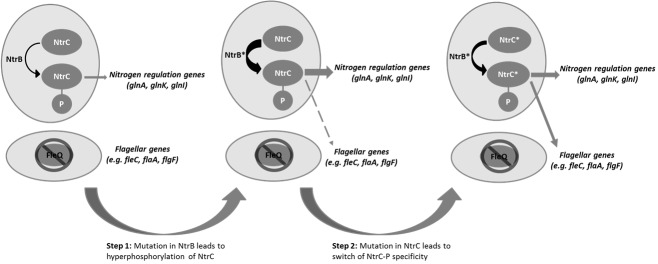
FIGURE 1: Proposed two-step evolutionary model for the resurrection of flagellar motility via rewiring of the nitrogen regulation pathway.

Both FleQ and NtrC are enhancer binding proteins (EBPs) and interact with RpoN (σ^54^) to activate transcription from certain promoters. The specificity of EBPs for their targets probably occurs through the enhancer binding HTH domain and in one sense it is perhaps no surprise that NtrC can be co-opted to fill in for the deficiency in the *fleQ *mutant. However, there are numerous other RpoN-dependent EBPs in *P. fluorescens*, some of which show closer structural homology to FleQ than NtrC (Figure 2). Why, then, was NtrC the only RpoN-dependent EBP to be co-opted in this way? A likely explanation for the repeatable rewiring of the nitrogen regulatory system in our study is because this adaptive trajectory is influenced by the ecological context. Under the conditions we tested, the immotile cells begin to starve, which we can predict causes intracellular nitrogen levels to fall and triggers increased phosphoryl transfer through the Ntr system. The cell is therefore primed with an active Ntr system.

**Figure 2:**
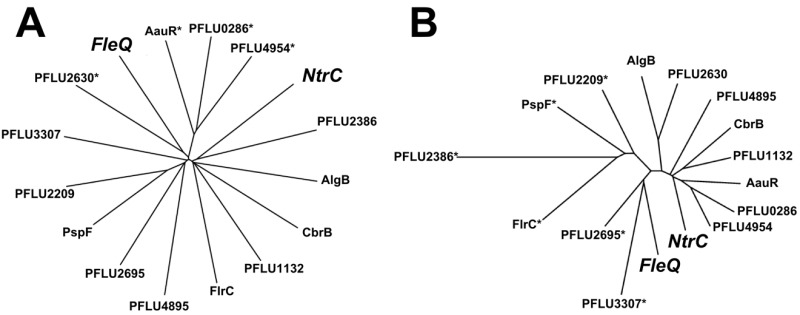
FIGURE 2: Star phylogenies showing comparison of the full length **(A) **protein sequence (Clustal Omega) and **(B)** predicted 3D structure (Neighbour joining) of RpoN-dependent EBPs in *P. fluorescens* SBW25. Proteins with the shortest distance travelled along lines are more similar. Note that several other proteins share closer similarity, both in sequence and structure, to FleQ (the most similar denoted by *).

Furthermore, starvation may mitigate the potential deleterious effects of mutations to the Ntr system. High nitrogen concentration is toxic to NtrB mutants; where local nitrogen is depleted, this severe constraint is removed. Starvation therefore provides a more favourable selective environment for the initial slow spreader, which constitutively overproduces NtrC-P, via mutation in NtrB, GlnA or GlnK. As NtrC-P levels increase, the titre of the protein is predicted to exceed levels required for the Ntr system and the *flg* genes are then activated by an overabundance of NtrC-P.

Intriguingly, our transcriptome data do not show activation of other RpoN-dependent genes outside of the *ntr* and *flg* systems, suggesting there is perhaps more potential for cross-talk between these two EBPs than originally appreciated. As the cells start moving from the point of inoculation, we then see the emergence of a faster moving sub-population of cells (*ntrC* mutants) arising from the slow moving mutant. The transcriptome data show that the *flg* genes in these strains are upregulated even further, to wild type levels of expression, while the *ntr* genes are tuned downwards. This confers faster motility affording reduced competition and improved access to unexploited resources, but may have an additional benefit; selection will favour mutants that are still able to move, but have reduced toxic effects of higher levels of nitrogen on the cell. A number of biochemical, genetic and ecological experiments are required to test these hypotheses.

An interesting question this raises is how NtrC-P is able to activate the *flg* genes in the slow and fast moving mutants. We propose two potential mechanisms that may be underpinning this switch in specificity: 1. NtrC-P binds onto *flg* enhancer sites; 2. NtrC-P activates *flg* genes by direct binding to RpoN. Although either or both mechanisms may be operating, our structural modelling of the proteins found that the *ntrC* mutations in the fast spreaders were located in, or close to, the DNA-binding HTH domain. The same point mutation (N454S) arose in four independent lines, suggesting that selection favoured a particular, precise alteration of binding specificity. However, one mutant had a gene deletion resulting in loss of the HTH domain; it is likely that in this case, the activated NtrC-P protein binds directly to RpoN at *flg* promoters. This raises the intriguing possibility that mutations in the same domain of the same protein could still potentially represent disparate evolutionary pathways to novel network function.

Finally, a further exciting question is to determine the evolutionary fate of the newly evolved fast mutant. Now that this strain lacks a key regulator but has evolved a dual functioning EBP, albeit without any environmental control, suggests that this strain should experience selective pressure to reinstate environmental control and perhaps evolve orthologous duplication to re-establish independent regulatory control.

Our knowledge of how regulatory systems function and adapt has been greatly improved by this discovery and this system can be used to examine how complex regulatory networks evolve. Francoise Jacob famously described evolution as a process of tinkering (*bricolage*), but this term has connotations of haphazard stochasticity that may be misleading, as evolutionary trajectories can be remarkably repeatable. It remains to be seen whether there will be continuing consistency in the longer-term trajectory of compensatory mutations, as NtrC further adapts to its dual roles following this major rewiring, or duplicates and specialises anew.

Further studies on the downstream physiological effects of this evolution will include analyses to determine how the adapted strain performs in different environments, particularly with regards to its modified capacity for nitrogen uptake, and especially under other ecological conditions and more complex environments. Verification of the immotile double mutants’ flagellar restoration ability under natural soil conditions will also be of interest to determine if such mutants can surmount the physiological disadvantage of losing regulatory control of flagella and nitrogen systems, to ensure better persistence when exposed to competing soil microorganisms and fluctuating nitrogen and carbon sources. Different *P. fluorescens* strains are often touted as highly promising biocontrol agents for various diseases, including, among others, fire blight, *Pythium* spp. and *Phytophthora* spp. diseases and take-all of wheat, as well as potent plant growth promoters. This evidence of a reproducible evolutionary capability to restore lost functions by adaptation shows promise for these bacteria as resilient biocontrol or biofertilizer treatments, able to adapt and maintain populations in changing conditions. A better understanding of how environmental and genetic factors affect motility in *P. fluorescens* could also increase knowledge of how and why soil colonization by these bacteria, and consequently biological activity, are often inconsistent during field application. This could lead to the improvement of efficiency and consistency of plant colonization by *P. fluorescens* treatments, ultimately leading to the reduced use of agrochemicals.

